# Glucocorticoid induces GSDMD-dependent pyrolysis in PC12 cells via endoplasmic reticulum stress

**DOI:** 10.1371/journal.pone.0274057

**Published:** 2022-09-01

**Authors:** Bin Yang, Tengteng Zhang, Lai Wei, Bin Zhao, Qingzhi Wang, Zhijun Yao, Shanyong Yi

**Affiliations:** 1 Xinxiang Key Laboratory of Forensic Toxicology, School of Forensic Medicine, Xinxiang Medical University, Xinxiang, Henan, China; 2 The Second Affiliated Hospital of Xinxiang Medical University, Henan Mental Hospital, Xinxiang, Henan, China; 3 Lifestyle Science Cluster, Advanced Medical and Dental Institute, Universiti Sains Malaysia, Pulau Pinang, Malaysia; 4 School of Basic Medical Science, Xinxiang Medical University, Xinxiang, Henan, China; Universidade de Sao Paulo, BRAZIL

## Abstract

**Objective:**

The present study explored whether pyroptosis is involved in the injury process of PC12 cells induced by glucocorticoid (GC) and the regulatory relationship between endoplasmic reticulum stress (ERS) and pyrolysis.

**Methods:**

LDH leakage of PC12 cells was detected by LDH assay. The number of dead cells was detected by SYTOX green nucleic acid staining. The levels of IL-1β and IL-18 in the supernatants was detected by ELSIA assay. The expression levels of glucose regulated protein 78 (GRP78), cleaved gasdermin D-NT (cleaved-GSDMD-NT), NLR-pyrin domain-containing 3 (NLRP3) and cleaved-caspase-1 were observed by immunofluorescence staining and western blot.

**Results:**

The LDH assay revealed that GC exposure significantly increased the release of LDH. The results of SYTOX green acid staining showed that GC exposure significantly increased the number of SYTOX green acid-positive cells. The ELSIA assay revealed that GC exposure significantly increased the levels of IL-1β and IL-18 in the supernatants. The results of immunofluorescence staining and western blot showed that GC exposure significantly increased the expression of GRP78, cleaved-GSDMD-NT, NLRP3 and cleaved caspase-1. Treatment with the ERS inhibitor tauroursodeoxycholate (TUDCA) and siRNA GSDMD attenuated related damage and downregulated the expression of the abovementioned proteins.

**Conclusion:**

The present study clearly demonstrated that GC exposure can induce GSDMD-dependent pyrolysis, and ERS is involved in the above damage process.

## 1. Introduction

As an important steroid hormone, GC is secreted by the adrenal cortex tract [1]. Physiological doses of GC can regulate material metabolism and maintain the body’s life activities [2]. When the body is stimulated by various internal and external factors, the hypothalamus pituitary adrenal (HPA) axis is activated, which can increase the synthesis and secretion of GC and produce anti-inflammatory, anti-infective, anti-shock, and immunosuppressive effects to maintain homeostasis [3]. Previous studies have shown that long-term high-intensity stress can induce the body to secrete a large amount of GC [4], and high-concentration GC can penetrate the blood-brain barrier, causing neuronal damage or even death and multiple mental disorders [5]. Cell models are efficient ways to identify the underlying mechanism of alcohol-induced neuronal apoptosis. As a cell line derived from rat adrenal pheochromocytoma, differentiated PC12 cells exhibit the general characteristics of neuroendocrine cells and are widely used in neurophysiology and neuropharmacology research. Our previous study has showed that high-concentration GC exposure can significantly inhibit the viability of PC12 cells and induce cell apoptosis. However, little is known regarding the mechanism of damage induced by GC.

The endoplasmic reticulum (ER) is an important organelle mainly responsible for protein synthesis, folding, and transport [6]. The correct and efficient folding of proteins plays an important role in cell survival and physiological functions [7]. Disruption of ER homeostasis can cause an accumulation of unfolded or misfolded proteins within the ER, which can activate ERS [8, 9]. However, persistent ERS can switch the cytoprotective function of the unfolded protein response (UPR) into cell death-promoting mechanisms [10]. There are three unique stress sensors located at the ER membrane: inositol-requiring protein 1α (IRE1α), PKR-like eukaryotic initiation factor 2α kinase (PERK), and activating transcription factor 6 (ATF6) [11]. Under normal physiological conditions, the above three transmembrane proteins all bind to the molecular chaperone GRP78 in an inactive state [12]. When ERS occurs, the accumulated unfolded or misfolded proteins competitively bind and sequester GRP78 away from PERK, IRE1, and ATF6. This leads to the activation of these three transducers, which in turn triggers a series of molecular reactions that cause various pathological damages, such as inflammation, apoptosis, and autophagy [13, 14]. Our previous study showed that the PERK-ATF4-CHOP and IRE1-ASK1-JNK pathways were involved in the GC-induced injury process of PC12 cells.

Pyroptosis is a type of programmed cell death [15]. As the executive protein of pyroptosis, gasdermins (GSDMs) are a recently characterized protein family encoded by six paralogous genes: gasdermin A (GSDMA), gasdermin B (GSDMB), gasdermin C (GSDMC), gasdermin D (GSDMD), gasdermin E (GSDME) and pejvakin (PJVK) [16]. GSDMs usually contain two protein functional domains (N-terminal and C-terminal domains), which form a conserved two-domain structure and share an autoinhibitory mechanism [17]. Under pathological conditions, GSDM-NT and GSDM-CT can be separated causing the autoinhibition effect to disappear. Cleaved-GSDM-NT can be connected to phospholipids on the cell membrane, resulting in their polymerization and insertion into the cell membrane to generate hole-like structures with an inner diameter of approximately 10–16 nm. As nonselective pores, GSDM pores are sufficient to allow the inflow of extracellular H_2_O and Ca^2+^ to cause cell swelling and disintegration; subsequently, the cell contents flow out, ultimately resulting in the release of IL-1β and IL-18 and pyroptosis [18].

However, whether pyroptosis is involved in the injury process of PC12 cells induced by glucocorticoid remains unclear as does the regulatory relationship between ERS and pyrolysis. For the present study, we aimed to investigate the mechanism of cell injury induced by GC at the cellular level and provide morphological evidence.

## 2. Materials and methods

### 2.1. Materials

The PC12 cell line was obtained from the American Type Culture Collection (Manassas, VA, USA). RPMI 1640 medium was purchased from Corning (Corning, NY, USA). Fetal bovine serum (FBS) was purchased from PAN Biotech (Aidenbach, Germany). Horse serum and nerve growth factor 2.5S (NGF 2.5S, 13257019) were purchased from Gibco (Grand Island, NY, USA). Sodium pyruvate solution and pen-strep solution were purchased from BI (Kibbutz Beit-Haemek, Israel). Opti-MEM medium was purchased from Invitrogen (Carlsbad, CA, United States). Short interfering RNAs (siRNAs) targeting GSDME (siRNA GSDMD), siRNA negative control (siRNA NC), and Lipofectamine 2000 ™ were purchased from RiboBio (Guangzhou, China). Dexamethasone (DEX) and tauroursodeoxycholate (TUDCA, commercially available glucocorticoids) were purchased from Sigma-Aldrich (St Louis, MO, USA). Cell freezing medium was purchased from ScienCell (Carlsbad, CA, USA). Lactate Dehydrogenase (LDH) assay kit was purchased from Solarbio Life Sciences (Beijing, China). SYTOX green acid staining solution was purchased from Invitrogen (Carlsbad, CA, USA). ELISA kit for IL-1β and IL-18 were purchased from Boster (Wuhan, Hubei, China). Rabbit antibodies against GRP78 (ab21685) and NLRP3 (ab270449) were purchased from Abcam (Cambridge, MA, USA). Rabbit antibody against cleaved-GSDMD-NT (#36425) was purchased from CST (Boston, MA, USA). Rabbit antibody against cleaved-caspase-1 (AF4022) was purchased from Affinity Biosciences (Melbourne, Australia). Rabbit antibody against β-actin (AF5003) was purchased from Beyotime (Shanghai, China). Alexa Fluor^™^ 594 donkey anti-rabbit IgG (H + L) (R37119) was purchased from Invitrogen (Carlsbad, CA, USA). All other chemicals and reagents used in the present study were analytical pure.

### 2.2. Cell culture and treatment

Cell culture and treatment were executed as described previously [19]. PC12 cells were cultured in RPMI 1640 medium supplemented with 7.5% fetal bovine serum, 5% horse serum, 100 U/mL penicillin/streptomycin, and 110 μg/mL sodium pyruvate solution at 37°C in a humidified atmosphere of 5% CO2. PC12 cells were incubated with 50 ng/mL NGF 2.5S for 48 h to induce neurite formation and imitate neurons. After seeding in appropriate petri dishes, differentiated PC12 cells were used for subsequent experiments.

For experiments involving siRNA transfections, PC12 cells were plated in 6-well plates to reach a confluence of 50% ~ 60%. Then transfections were fulfilled using 5μl Lipofectamine 2000™ added to 250μl of Opti-MEM medium, which was incubated at room temperature for 5 min. In another tube, 5μl of 50nM siRNAs were added into 250μl Opti-MEM medium before both tubes were mixed and incubated at room temperature for 20 min. Cells were incubated in this mixture for 8 hours.

### 2.3. LDH release assay

Briefly, PC12 cells were treated with 100 μM DEX for 24 h. Then, supernatants were extracted and treated with an LDH assay kit. Finally, treated samples were detected at 450 nm by Multiskan Go (Waltham, MA, USA).

In LDH release assay, the experiments set the following four groups: control, DEX (100 μM), DEX + TUDCA (DEX: 100 μM; TUDCA: antagonist for endoplasmic reticulum stress, 100 μM), and TUDCA (100 μM).

### 2.4. SYTOX green nucleic acid staining

PC12 cells were treated with 100 μM DEX for 24 h. Cells were washed three times in Hank’s balanced salt solution (HBSS) and incubated with SYTOX green acid staining solution for 20 min in the dark. Then, the cells were washed three times in HBSS and images were acquired by a confocal laser scanning microscope (Leica, Ernst-Leitz-Strasse, Wetzlar, Germany).

In SYTOX green nucleic acid staining, the experiments set the following four groups: control, DEX (100 μM), DEX + TUDCA (DEX: 100 μM; TUDCA: antagonist for endoplasmic reticulum stress, 100 μM), and TUDCA (100 μM).

### 2.5. ELISA assay

Briefly, PC12 cells were treated with 100 μM DEX for 24 h. Then, supernatants were extracted and treated according to the ELISA kit. Finally, treated samples were detected at 450 nm by Multiskan Go (Waltham, MA, USA).

In ELISA assay, the experiments set the following four groups: control, DEX (100 μM), DEX + TUDCA (DEX: 100 μM; TUDCA: antagonist for endoplasmic reticulum stress, 100 μM), and TUDCA (100 μM).

### 2.6. Immunofluorescence staining

Immunofluorescence was performed as described previously [19]. Cells were fixed with 4% paraformaldehyde at room temperature for 15 min, incubated with 0.2% Triton X-100 for 10 min on ice and then incubated in goat serum for 30 min. Next, the cells were incubated overnight at 4°C with antibodies specific for rabbit GRP78 (1:200), cleaved GSDMD-NT (1:200), NLRP3 (1:100) and cleaved-caspase-1(1:200), then incubated at 37°C with Alexa FluorTM 594 donkey anti-rabbit IgG (H+L). Finally, the cells were counterstained with DAPI and images were acquired by a confocal laser scanning microscope (Leica, Ernst-Leitz-Strasse, Wetzlar, Germany).

In the present study, Image-Pro Plus 5.1 (Media Cybernetics, Houston, TX, USA) was used to count the number of cells and detect the fluorescence intensity (IOD) of all cells. The average IOD was used to illustrate the expression levels of the corresponding proteins.

In immunofluorescence staining, the experiments set the following four groups: control, DEX (100 μM), DEX + TUDCA (DEX: 100 μM; TUDCA: antagonist for endoplasmic reticulum stress, 100 μM), and TUDCA (100 μM).

### 2.7. Western blot

Total protein (50 μg of protein/lane) was put into SDS–PAGE gels, then detached by electrophoresis and delivered to PVDF membranes. Subsequently, the target areas of the membranes were hatched with rabbit antibodies for GRP78 (1:1000), cleaved GSDMD-NT (1:1000) and β-actin (1:2000) overnight at 4°C. Then, the target areas of the membranes were hatched with goat anti-rabbit IgG (H+L) secondary antibody (#31210, Invitrogen, CA, USA) and detected by X-ray film with an enhanced chemiluminescence system. ImageJ 1.48 (National Institutes of Health, Maryland, USA) was applied to investigate the intensity of the bands.

In western blot of Fig 3, the experiments set the following four groups: siRNA NC (siRNA NC: 5μl of 50nM), DEX + siRNA NC (DEX:100 μM; siRNA NC: 5μl of 50nM), DEX + siRNA GSDMD (DEX: 100 μM; siRNA GSDMD: 5μl of 50nM), and siRNA GSDMD (siRNA GSDMD: 5μl of 50nM).

In western blot of Fig 8, the experiments set the following four groups: control, DEX (100 μM), DEX + TUDCA (DEX: 100 μM; TUDCA: antagonist for endoplasmic reticulum stress, 100 μM), and TUDCA (100 μM).

### 2.8. Statistical methods

The results are presented as mean ± SEM. Data were analyzed by one-way ANOVA followed by a post hoc least significant difference (LSD) t-test to determine specific group differences, and the effects of TUDCA and siRNA GSDMD were analyzed by two-way ANOVA. All statistical analyses were performed using SPSS 21.0. Significance was defined as *P* < 0.05 for all statistical tests.

## 3. Results

### 3.1. LDH leakage of PC12 cells

**Fig 1:**
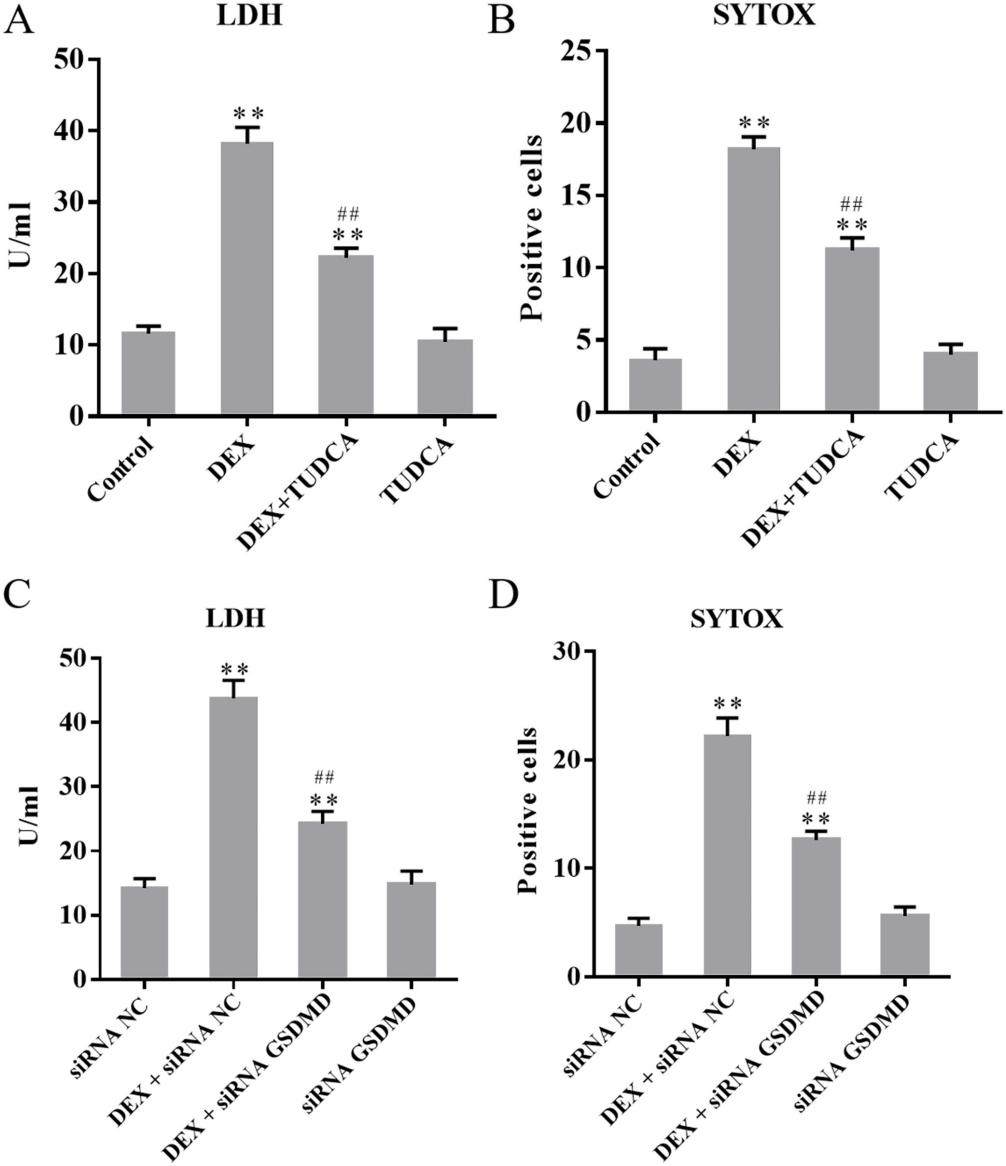
(A) The effect of DEX on the LDH leakage of PC12 cells (n = 5). (B) The effect of DEX on the number of SYTOX positive cells (n = 5). (C) The effect of DEX on the LDH leakage of PC12 cells (n = 5). (D) The effect of DEX on the number of SYTOX positive cells (n = 5). The release of LDH and the number of SYTOX green acid-positive cells were significantly increased after treatment with DEX, and TUDCA and siRNA GSDMD can significantly alleviate the effects of DEX. The data are shown as the mean ± SEM, ^**^P < 0.01 compared to the control group; ^##^*P* < 0.01 compared to the DEX group, ^**^P < 0.01 compared to the siRNA NC group; ^##^*P* < 0.01 compared to the DEX + siRNA NC group. DEX: dexamethasone; TUDCA: tauroursodeoxycholate; GSDMD: Gasdermin D.

Compared to the control group (11.60 ± 1.08), the LDH leakage of PC12 cells remained at a low level in the TUDCA group (10.40 ± 1.91, *P* > 0.05) and was significantly increased in the DEX group (38.20 ± 2.31, *P* < 0.01) and DEX + TUDCA group (22.20 ± 1.39, *P* < 0.01). Compared to the DEX group, the LDH leakage of PC12 cells was significantly decreased in the DEX + TUDCA group (*P* < 0.01) (Fig 1A).

Compared to the siRNA NC group (14.2 ± 1.50), the LDH leakage of PC12 cells remained at a low level in the siRNA GSDMD group (14.8 ± 2.06, *P* > 0.05) and was significantly increased in the DEX + siRNA NC group (43.8 ± 2.75, *P* < 0.01) and DEX + siRNA GSDMD group (24.20 ± 1.99, *P* < 0.01). Compared to the DEX + siRNA NC group, the LDH leakage of PC12 cells was significantly decreased in the DEX + siRNA GSDMD group (*P* < 0.01) (Fig 1C).

### 3.2. The number of SYTOX positive cells

Compared to the control group (3.6 ± 0.81), the number of SYTOX positive cells remained at a low level in the TUDCA group (4.0 ± 0.71, *P* > 0.05) and was significantly increased in the DEX group (18.20 ± 0.86, *P* < 0.01) and DEX + TUDCA group (11.20 ± 0.87, *P* < 0.01). Compared to the DEX group, the number of SYTOX positive cells was significantly decreased in the DEX + TUDCA group (*P* < 0.01) (Fig 1B).

Compared to the siRNA NC group (4.7 ± 0.70), the number of SYTOX positive cells remained at a low level in the siRNA GSDMD group (5.6 ± 0.83, *P* > 0.05) and was significantly increased in the DEX + siRNA NC group (22.2 ± 1.67, *P* < 0.01) and DEX + siRNA GSDMD group (12.6 ± 0.82, *P* < 0.01). Compared to the DEX + siRNA NC group, the number of SYTOX positive cells was significantly decreased in the DEX + siRNA GSDMD group (*P* < 0.01) (Fig 1D).

### 3.3. The levels of IL-1β and IL-18 in the supernatants

**Fig 2:**
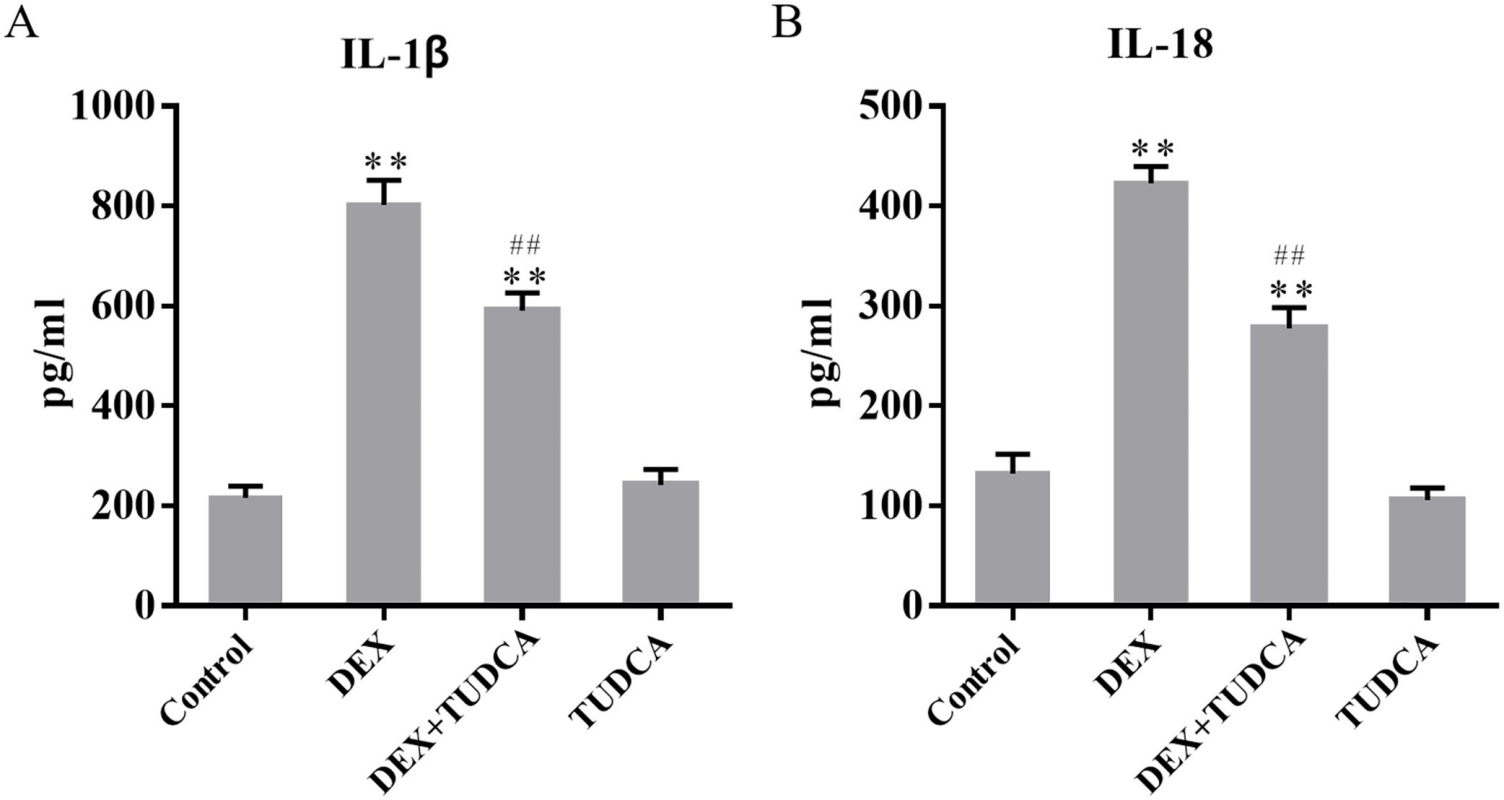
(A) The effect of DEX on the level of IL-1β in the supernatants (n = 5). (B) The effect of DEX on the level of IL-18 in the supernatants (n = 5). The level of IL-1β and IL-18 were significantly increased after treatment with DEX, and TUDCA can significantly alleviate the effects of DEX. ^**^P < 0.01 compared to the control group; ^##^*P* < 0.01 compared to the DEX group. DEX: dexamethasone; TUDCA: tauroursodeoxycholate.

Compared to the control group (216.20 ± 23.15), the level of IL-1β remained at a low level in the TUDCA group (241.80 ± 31.62, *P* > 0.05) and was significantly increased in the DEX group (801.80 ± 50.01, *P* < 0.01) and DEX + TUDCA group (590.60 ± 35.64, *P* < 0.01). Compared to the DEX group, the level of IL-1β was significantly decreased in the DEX + TUDCA group (*P* < 0.01) (Fig 2A).

Compared to the control group (132.40 ± 19.41), the level of IL-18 remained at a low level in the TUDCA group (106.00 ± 12.13, *P* > 0.05) and was significantly increased in the DEX group (422.60 ± 16.81, *P* < 0.01) and DEX + TUDCA group (277.60 ± 21.08, *P* < 0.01). Compared to the DEX group, the level of IL-18 was significantly decreased in the DEX + TUDCA group (*P* < 0.01) (Fig 2B).

### 3.4. Cleaved GSDMD-NT protein expression in PC12 cells by western blot

**Fig 3:**
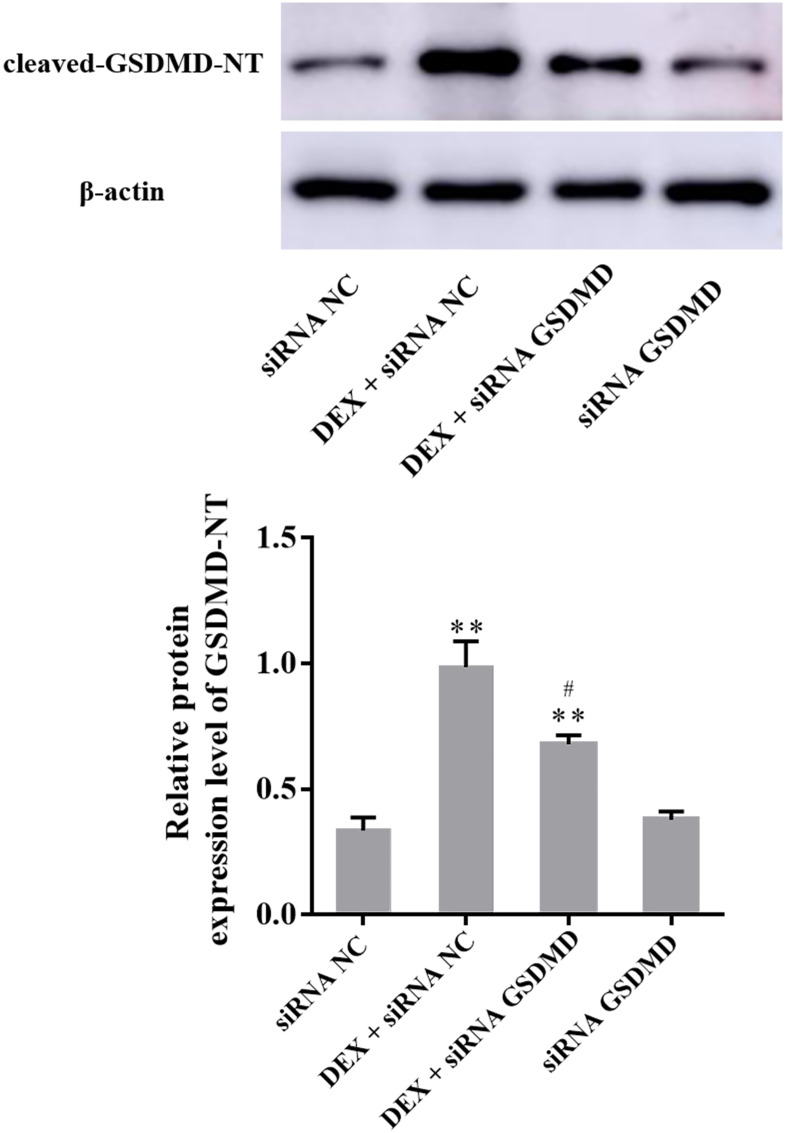
Western blot showed the expression level of cleaved GSDMD-NT in PC12 cells (n = 3). The expression level of cleaved GSDMD-NT was significantly increased after treatment with DEX, and siRNA GSDMD can significantly alleviate the effects of DEX. The data are shown as the mean ± SEM, ^**^P < 0.01 compared to the siRNA NC group; ^#^*P* < 0.05 compared to the DEX + siRNA NC group. GSDMD: Gasdermin D; DEX: dexamethasone.

Compared to the siRNA NC group (0.34 ± 0.05), the expression of cleaved GSDMD-NT remained at a low level in the siRNA GSDMD group (0.38 ± 0.03, *P* > 0.05) and was significantly increased in the DEX + siRNA NC group (0.99 ± 0.10, *P* < 0.01) and DEX + siRNA GSDMD group (0.68 ± 0.04, *P* < 0.01). Compared to the DEX + siRNA NC group, the expression of cleaved GSDMD-NT was significantly decreased in the DEX + siRNA GSDMD group (*P* < 0.05) (Fig 3).

### 3.5. GRP78, cleaved GSDMD-NT, NLRP3 and cleaved caspase-1 protein expression in PC12 cells by immunohistochemistry

**Fig 4:**
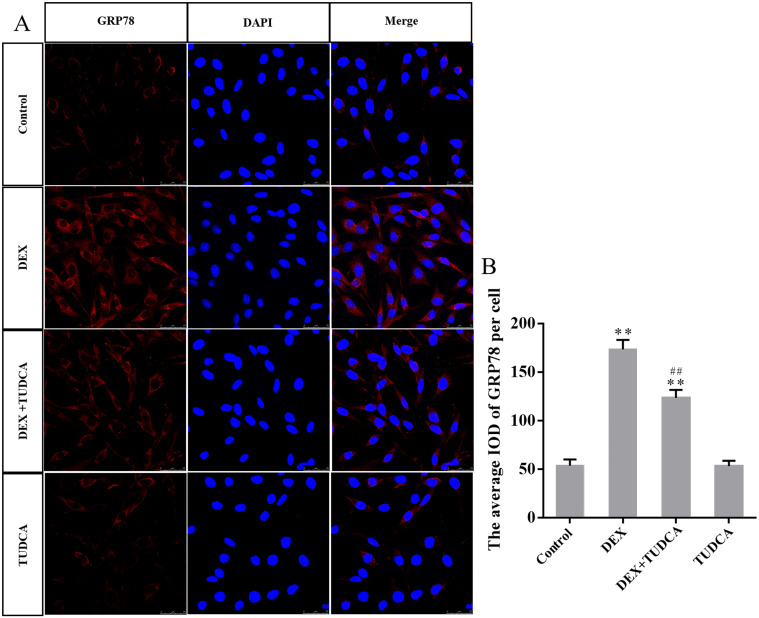
(A) Representative images showing GRP78 immunofluorescence in PC12 cells. (B) The average IOD of GRP78 per cell (n = 3). Bars = 25μm (magnification: × 800). The expression level of GRP78 was significantly increased after treatment with DEX, and TUDCA can significantly alleviate the effects of DEX. The data are shown as the mean ± SEM, ^**^P < 0.01 compared to the control group; ^##^*P* < 0.01 compared to the DEX group. GRP78: glucose-regulated protein 78; DEX: dexamethasone; TUDCA: tauroursodeoxycholate.

Compared to the control group (53.40 ± 6.52), the expression of GRP78 remained at a low level in the TUDCA group (53.00 ± 5.62, *P* > 0.05) and was significantly increased in the DEX group (173.40 ± 9.68, *P* < 0.01) and DEX + TUDCA group (123.40 ± 8.21, *P* < 0.01). Compared to the DEX group, the expression of GRP78 was significantly decreased in the DEX + TUDCA group (*P* < 0.01) (Fig 4).

**Fig 5:**
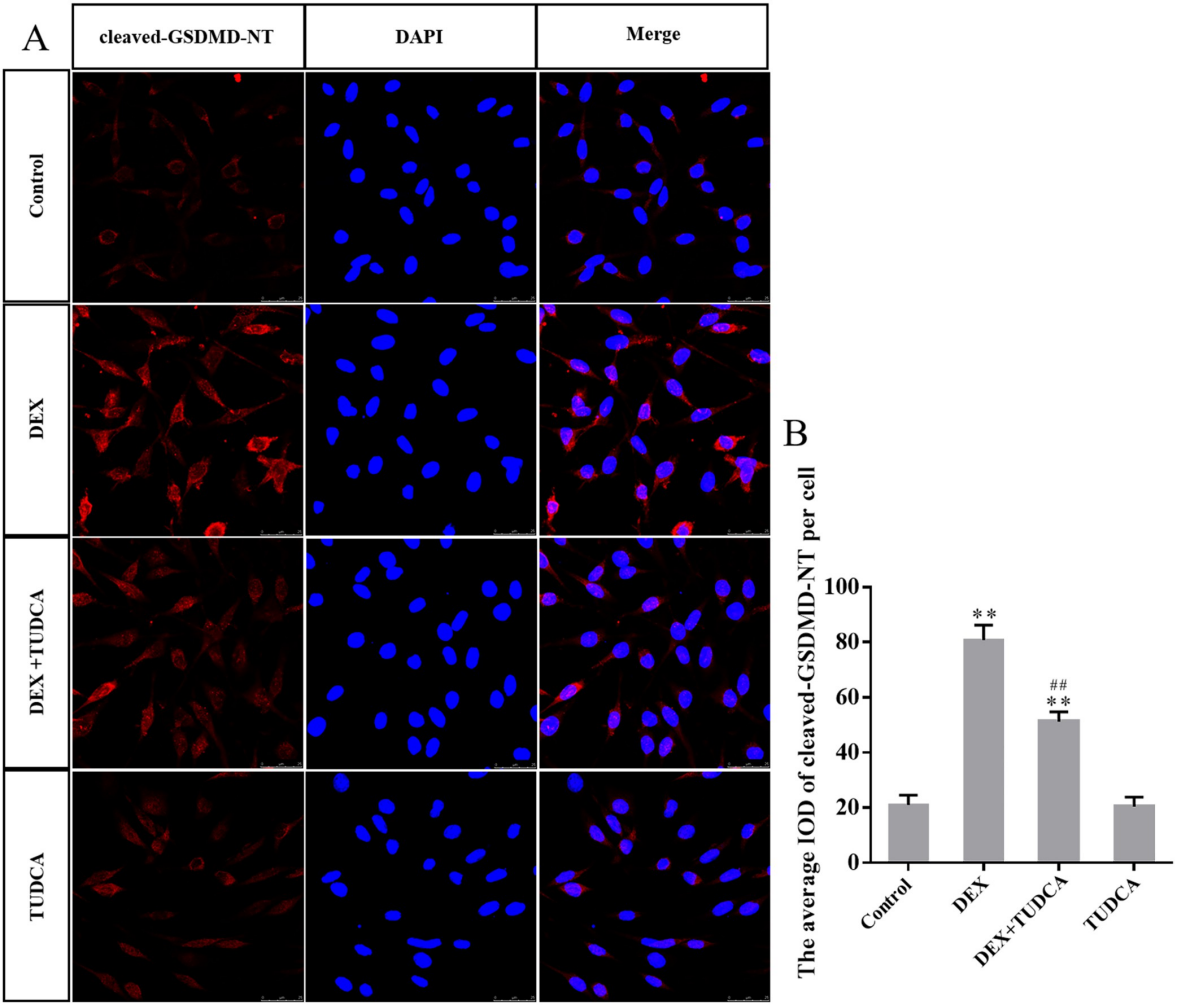
(A) Representative images showing cleaved-GSDMD-NT immunofluorescence in PC12 cells. (B) The average IOD of cleaved GSDMD-NT per cell (n = 3). Bars = 25μm (magnification: × 800). The expression level of GSDMD-NT was significantly increased after treatment with DEX, and TUDCA can significantly alleviate the effects of DEX. The data are shown as the mean ± SEM, ^**^P < 0.01 compared to the control group; ^##^*P* < 0.01 compared to the DEX group. GSDMD: Gasdermin D; DEX: dexamethasone; TUDCA: tauroursodeoxycholate.

Compared to the control group (21.00 ± 3.51), the expression of cleaved GSDMD-NT remained at a low level in the TUDCA group (20.40 ± 3.49, *P* > 0.05) and was significantly increased in the DEX group (80.80 ± 5.43, *P* < 0.01) and DEX + TUDCA group (51.20 ± 3.56, *P* < 0.01). Compared to the DEX group, the expression of cleaved GSDMD-NT was significantly decreased in the DEX + TUDCA group (*P* < 0.01) (Fig 5).

**Fig 6:**
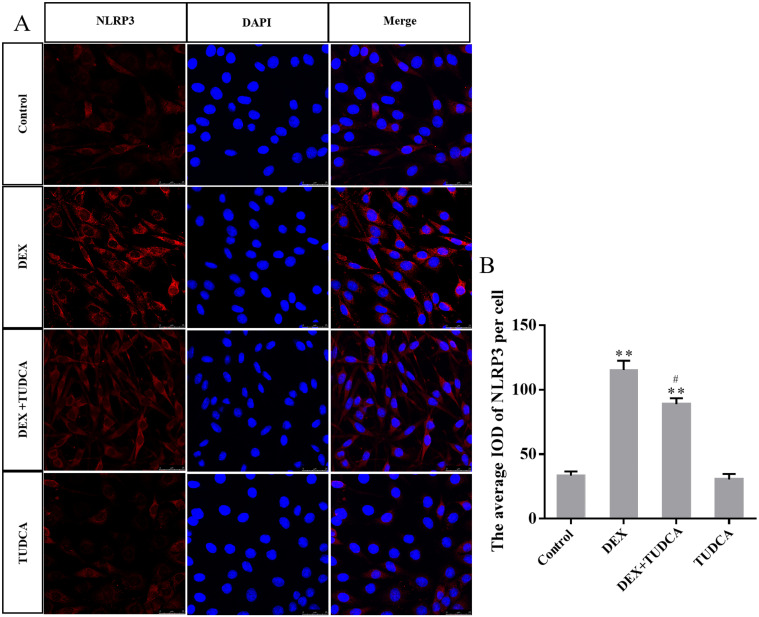
(A) Representative images showing NLRP3 immunofluorescence in PC12 cells. (B) The average IOD of NLRP3 per cell (n = 3). Bars = 25μm (magnification: × 800). The expression level of NLRP3 was significantly increased after treatment with DEX, and TUDCA can significantly alleviate the effects of DEX. The data are shown as the mean ± SEM, ^**^P < 0.01 compared to the control group; ^#^*P* < 0.05 compared to the DEX group. NLRP3: NLR-pyrin domain-containing 3; DEX: dexamethasone; TUDCA: tauroursodeoxycholate.

Compared to the control group (33.40 ± 3.23), the expression of NLRP3 remained at a low level in the TUDCA group (30.40 ± 4.40, *P* > 0.05) and was significantly increased in the DEX group (115.20 ± 7.38, *P* < 0.01) and DEX + TUDCA group (88.80 ± 4.63, *P* < 0.01). Compared to the DEX group, the expression of NLRP3 was significantly decreased in the DEX + TUDCA group (*P* < 0.05) (Fig 6).

**Fig 7:**
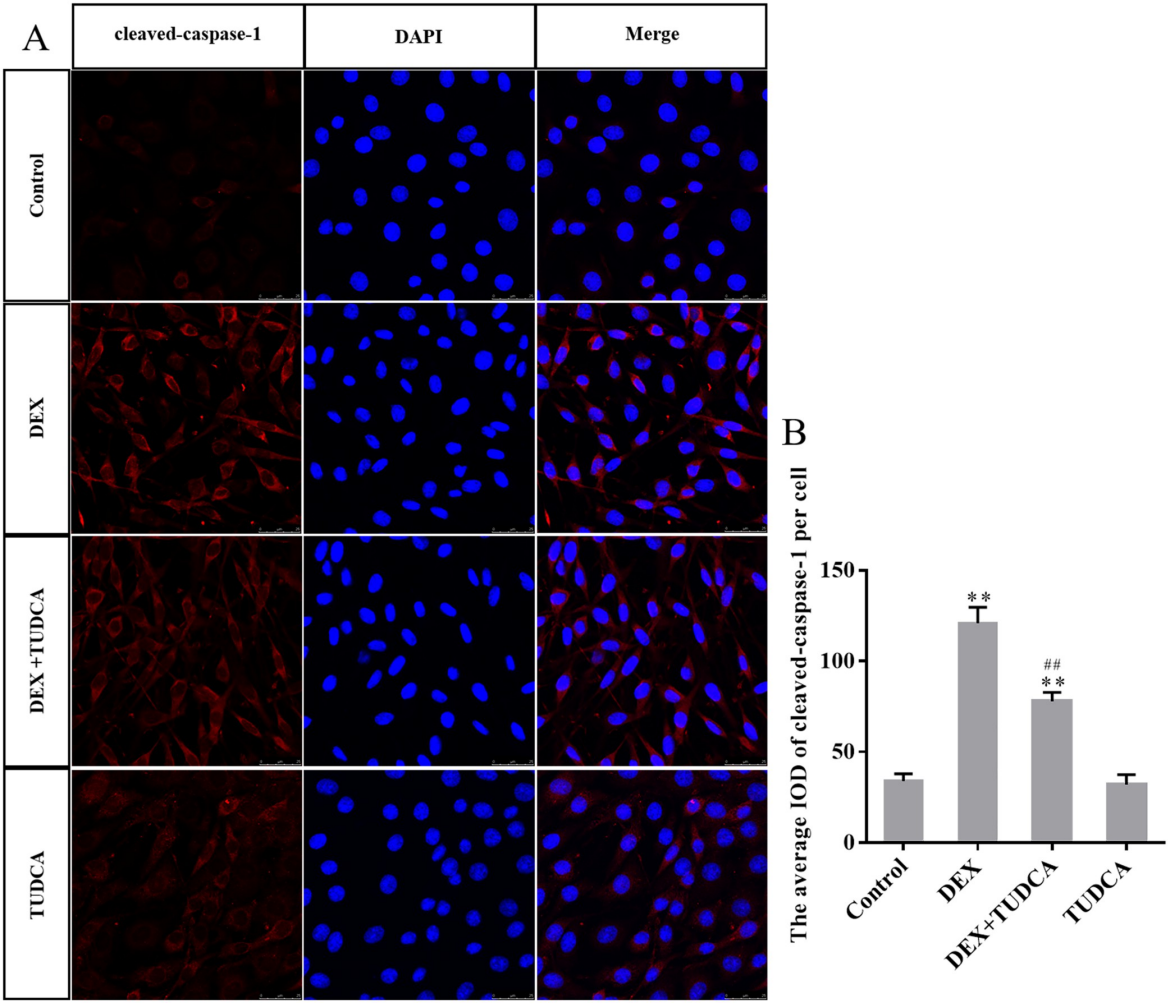
(A) Representative images showing cleaved-caspase-1 immunofluorescence in PC12 cells. (B) The average IOD of cleaved-caspase-1 per cell (n = 3). Bars = 25μm (magnification: × 800). The expression level of cleaved-caspase-1 was significantly increased after treatment with DEX, and TUDCA can significantly alleviate the effects of DEX. The data are shown as the mean ± SEM, ^**^P < 0.01 compared to the control group; ^##^*P* < 0.01 compared to the DEX group. DEX: dexamethasone; TUDCA: tauroursodeoxycholate.

Compared to the control group (34.00 ± 4.00), the expression of cleaved caspase-1 remained at a low level in the TUDCA group (32.00 ± 5.40, *P* > 0.05) and was significantly increased in the DEX group (121.00 ± 8.71, *P* < 0.01) and DEX + TUDCA group (77.80 ± 5.01, *P* < 0.01). Compared to the DEX group, the expression of cleaved-caspase-1 was significantly decreased in the DEX + TUDCA group (*P* < 0.01) (Fig 7).

### 3.6. GRP78 and cleaved GSDMD-NT protein expression in PC12 cells by western blot

**Fig 8:**
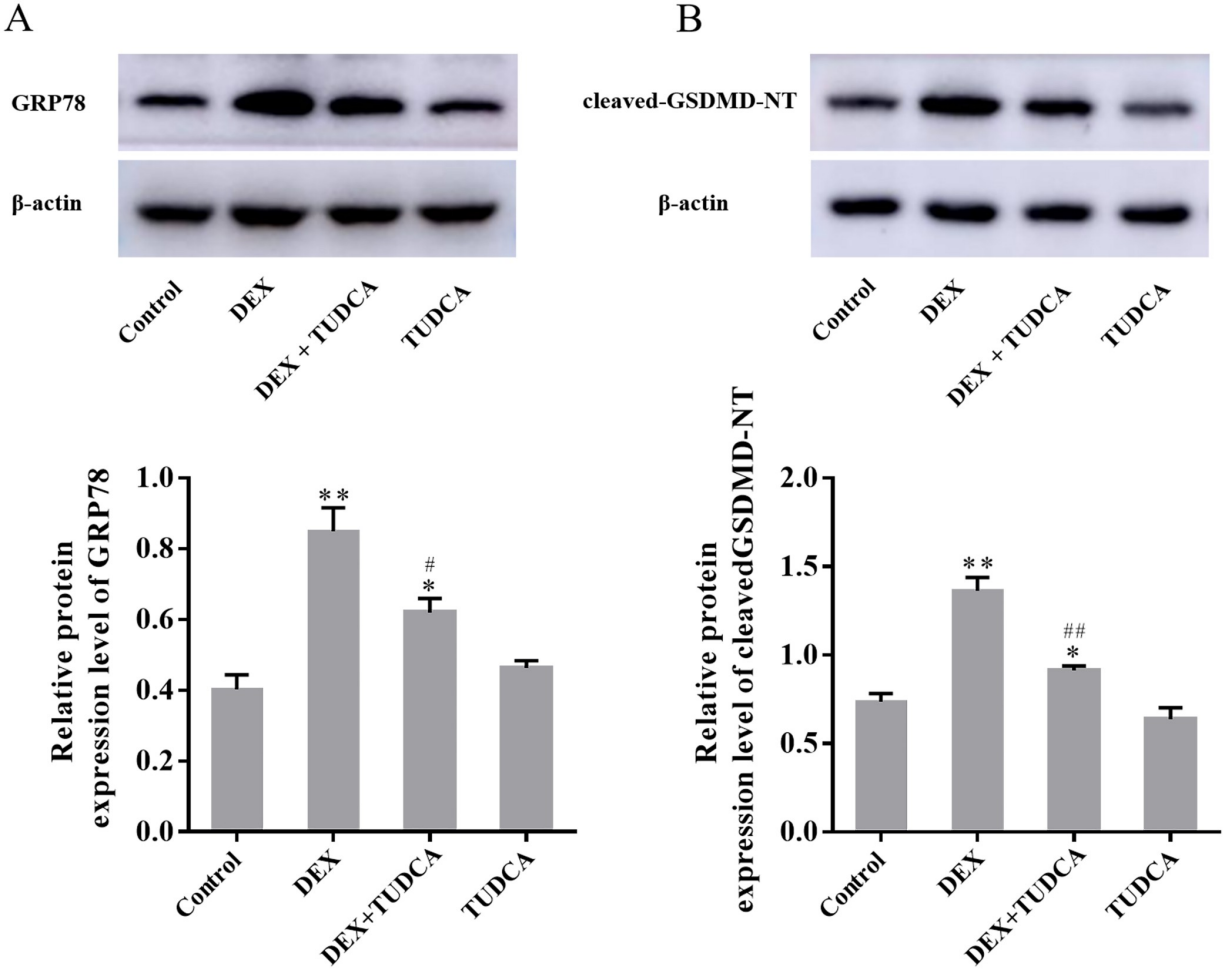
(A) Western blot showed the expression level of GRP78 in PC12 cells (n = 3). (B) Western blot showed the expression level of cleaved GSDMD-NT in PC12 cells (n = 3). The expression levels of GRP78 and cleaved GSDMD-NT were significantly increased after treatment with DEX, and TUDCA can significantly alleviate the effects of DEX. The data are shown as the mean ± SEM, ^*^P < 0.05 and ^**^P < 0.01 compared to the control group; ^#^*P* < 0.05 and ^##^*P* < 0.01compared to the DEX group. GSDMD: Gasdermin D; DEX: dexamethasone; TUDCA: tauroursodeoxycholate.

Compared to the control group (0.40 ± 0.04), the expression of GRP78 remained at a low level in the TUDCA group (0.46 ± 0.02, *P* > 0.05) and was significantly increased in the DEX group (0.85 ± 0.07, *P* < 0.01) and DEX + TUDCA group (0.62 ± 0.04, *P* < 0.05). Compared to the DEX group, the expression of GRP78 was significantly decreased in the DEX + TUDCA group (*P* < 0.05) (Fig 8A).

Compared to the control group (0.74 ± 0.05), the expression of cleaved GSDMD-NT remained at a low level in the TUDCA group (0.64 ± 0.07, *P* > 0.05) and was significantly increased in the DEX group (1.36 ± 0.08, *P* < 0.01) and DEX + TUDCA group (0.91 ± 0.03, *P* < 0.05). Compared to the DEX group, the expression of cleaved GSDMD-NT was significantly decreased in the DEX + TUDCA group (*P* < 0.01) (Fig 8B).

## 4. Discussion

The previous study showed that PC12 cells have obvious characteristics of neurons after being treated with NGF and abundantly express glucocorticoid receptors [19], which indicates that PC12 cells are suitable for investigating the mechanism of GC-induced neuronal injury. The physiological dose of GC can regulate material metabolism and has anti-inflammatory, anti-infective, anti-shock, and immunosuppressive effects to maintain homeostasis [3]. However, research indicates that continuous stress causes excessive secretion of GC, which can infiltrate the blood-brain barrier and induce neuronal injury [5]. Our previous study showed that the viability of PC12 cells is significantly reduced and the rate of apoptosis is significantly increased after treatment with 100 μM DEX for 24 h [19]. In the present study, the results of LDH assays showed that GC exposure can significantly increase the release of LDH. The results of SYTOX green acid staining showed that GC exposure can significantly increase the number of SYTOX green acid-positive cells. Also, the results of ELISA assay showed that GC exposure can significantly increase the levels of IL-1β and IL-18 in the supernatants. The above results showed that GC can significantly damage the cell membrane and increase the leakage of cell contents, indicating that pyrolysis may be involved in the process of PC12 cell damage induced by GC.

Pyroptosis, also known as cell inflammatory necrosis, is a kind of programmed cell death [20]. Among the six protein subtypes of pyrolysis, the GSDMD-related classical pyrolysis pathway has been fully studied [17]. As a member of the nucleotide-binding domain like receptor (NLR) family, NLRP3 can activate the inflammasome and induce pyroptosis [21]. The NLRP3 inflammasome is composed of NLRP3, apoptosis associated speck-like protein containing a CARD (ASC), and proinflammatory caspase-1 [22]. When cells are subjected to various internal and external stimuli, NLRP3 oligomerizes and exposes pyrin domains (PYDs), which can interact with ASCs. Then, the CARDs of ASCs interact with pro-caspase-1, which can enable caspase-1 activation [23]. Cleavage of GSDMD is achieved by caspase family proteins. Caspase-1 and caspase-11 (caspase-4/5 in humans) can recognize and cleave GSDMD to form cleaved GSDMD-NT, which can induce pyrolysis [24]. In the present study, the expression levels of NLRP3, cleaved caspase-1 and cleaved GSDMD-NT were significantly increased after treatment with DEX, while after treating PC12 cells with siRNA GSDMD, the expression of cleaved GSDMD-NT was significantly decreased, the release of LDH and the number of SYTOX green acid-positive cells were also significantly reduced, which indicating that the NLRP3/caspase-1/GSDMD pathway was involved in pyrolysis of PC12 cells induced by GC.

As a widely distributed membranous organelle, the ER is responsible for the synthesis, folding, and transport of most proteins in eukaryotic cells [25]. Unfolded or misfolded proteins can lead to the accumulation of abnormal proteins in the cell, which then activates the UPR and leads to ERS [26]. Under ERS conditions, the expression of GRP78 is significantly upregulated, and is often used as an activation marker of ERS [27]. In the present study, the expression of GRP78 was significantly increased after treatment with DEX, indicating that GC can induce ERS in PC12 cells. Application of the ERS inhibitor TUDCA significantly reduced the release of LDH and the number of SYTOX green acid-positive cells, the levels of IL-1β and IL-18 in the supernatants and the expression levels of GRP78, NLRP3, cleaved caspase-1, and cleaved-GSDMD-NT were also significantly decreased. The above results suggest that ERS was involved in the GC-induced pyrolysis of PC12 cells via the NLRP3/caspase-1/GSDMD pathway.

In conclusion, the present study clearly demonstrated that GC exposure can induce GSDMD-dependent pyrolysis, and ERS is involved in the above damage process. We believe these novel findings will provide a new research perspective on the mechanism of GC-induced neuronal injury.

## Supporting information

S1 Raw images (PDF)
