# Supplementary material for: Glucocorticoid induces GSDMD-dependent pyrolysis in PC12 cells via endoplasmic reticulum stress
Source: PLoS One. 2022 Sep 1;17(9):e0274057. doi: 10.1371/journal.pone.0274057 (PMC9436126; doi:10.1371/journal.pone.0274057)

The original blots of cleaved GSDMD-NT in Fig.3

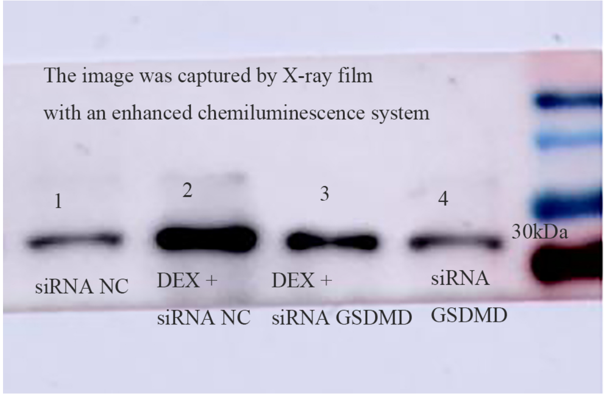

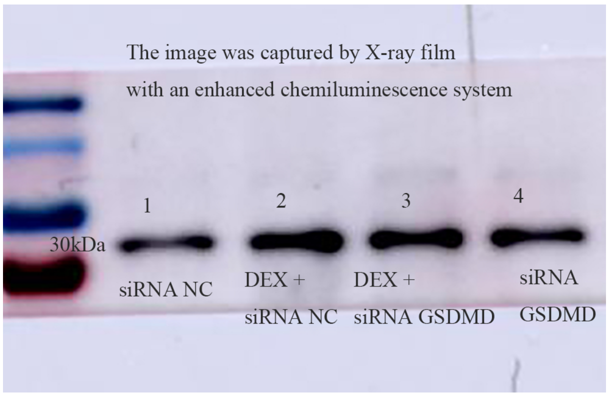

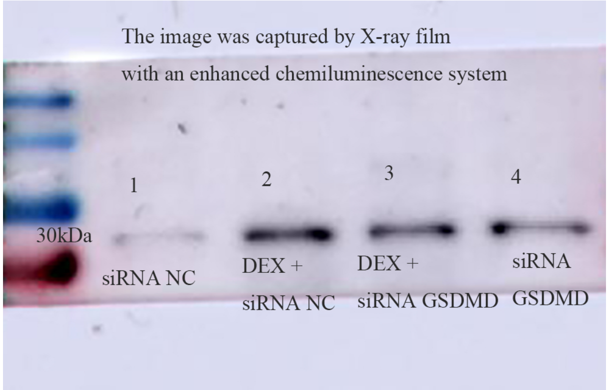

The original blots of β-actin in Fig.3

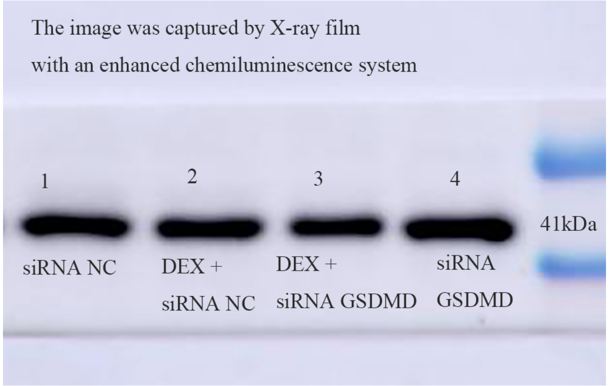

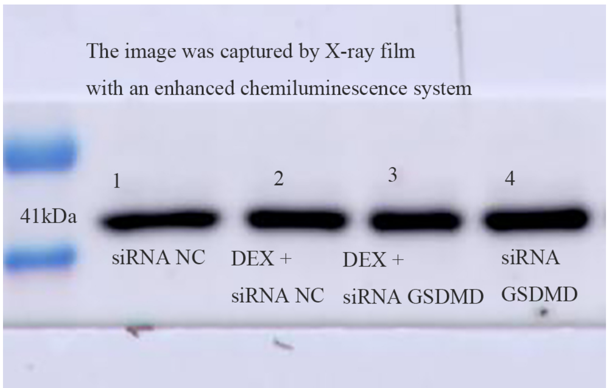

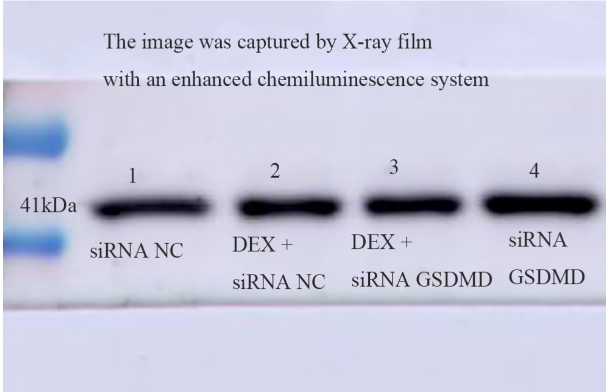

The original blots of GRP78 in Fig.8A

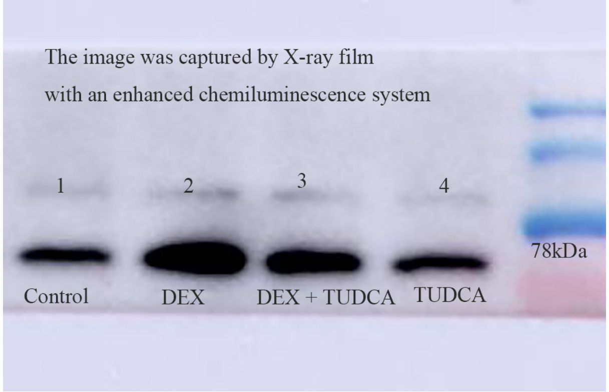

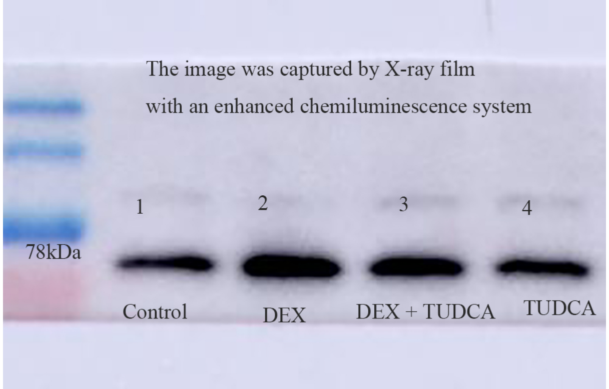

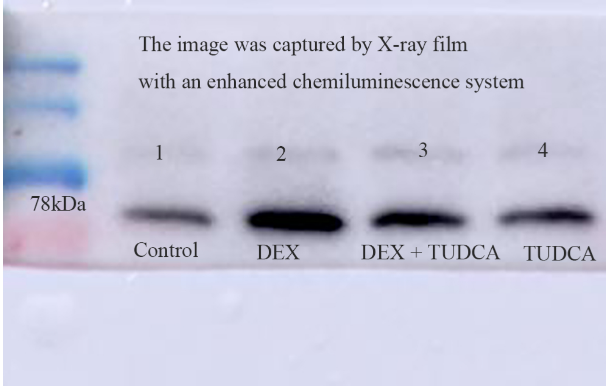

The original blots of β-actin in Fig.8A

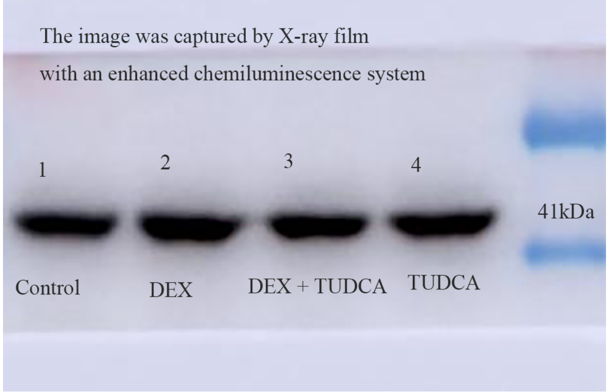

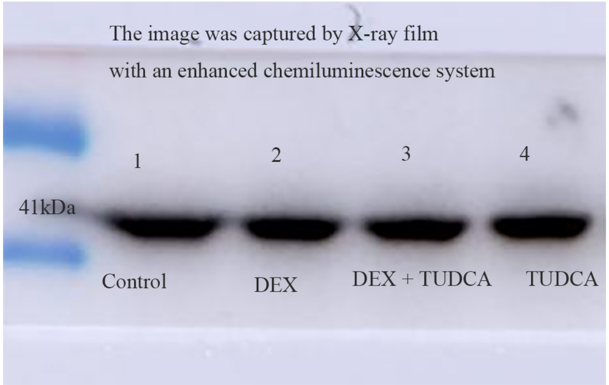

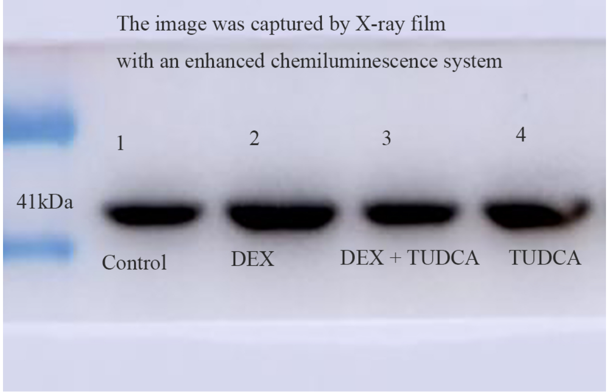

The original blots of cleaved GSDMD-NT in Fig.8B

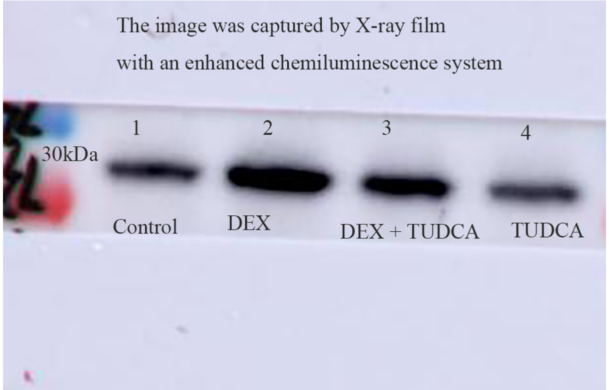

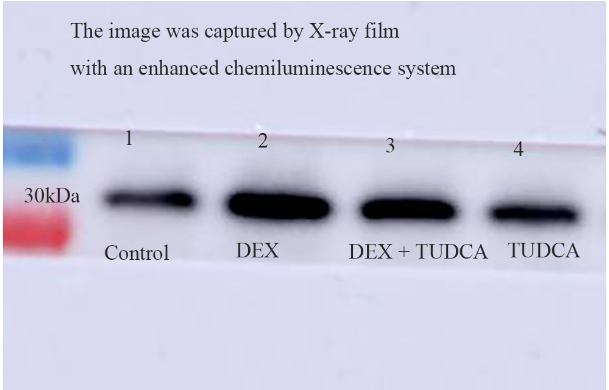

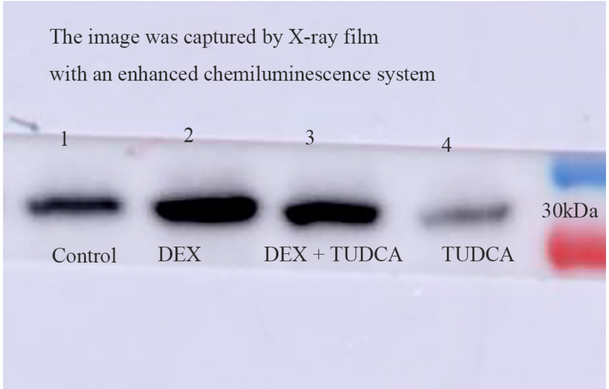

The original blots of β-actin in Fig.8B

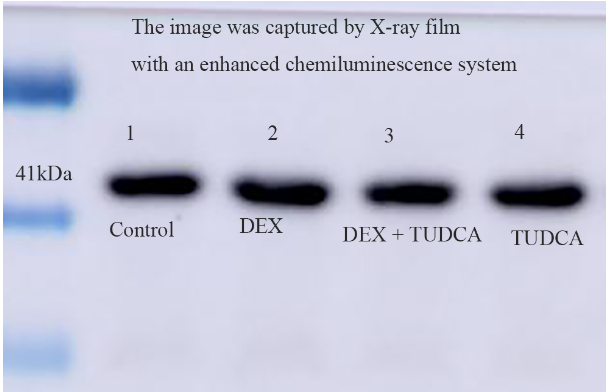

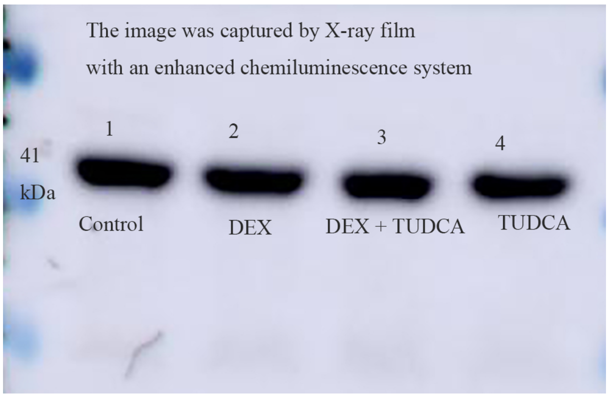

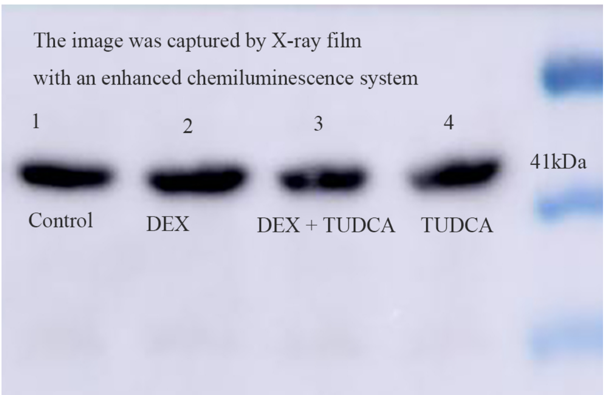

Supplement: S1 Raw images — (PDF) [file pone.0274057.s001.pdf]
